# Supplementary figures and images for: Can endocranial volume be estimated accurately from external skull measurements in great-tailed grackles (Quiscalus mexicanus)?
Source: PeerJ. 2015 Jun 11;3:e1000. doi: 10.7717/peerj.1000 (PMC4465945; doi:10.7717/peerj.1000)

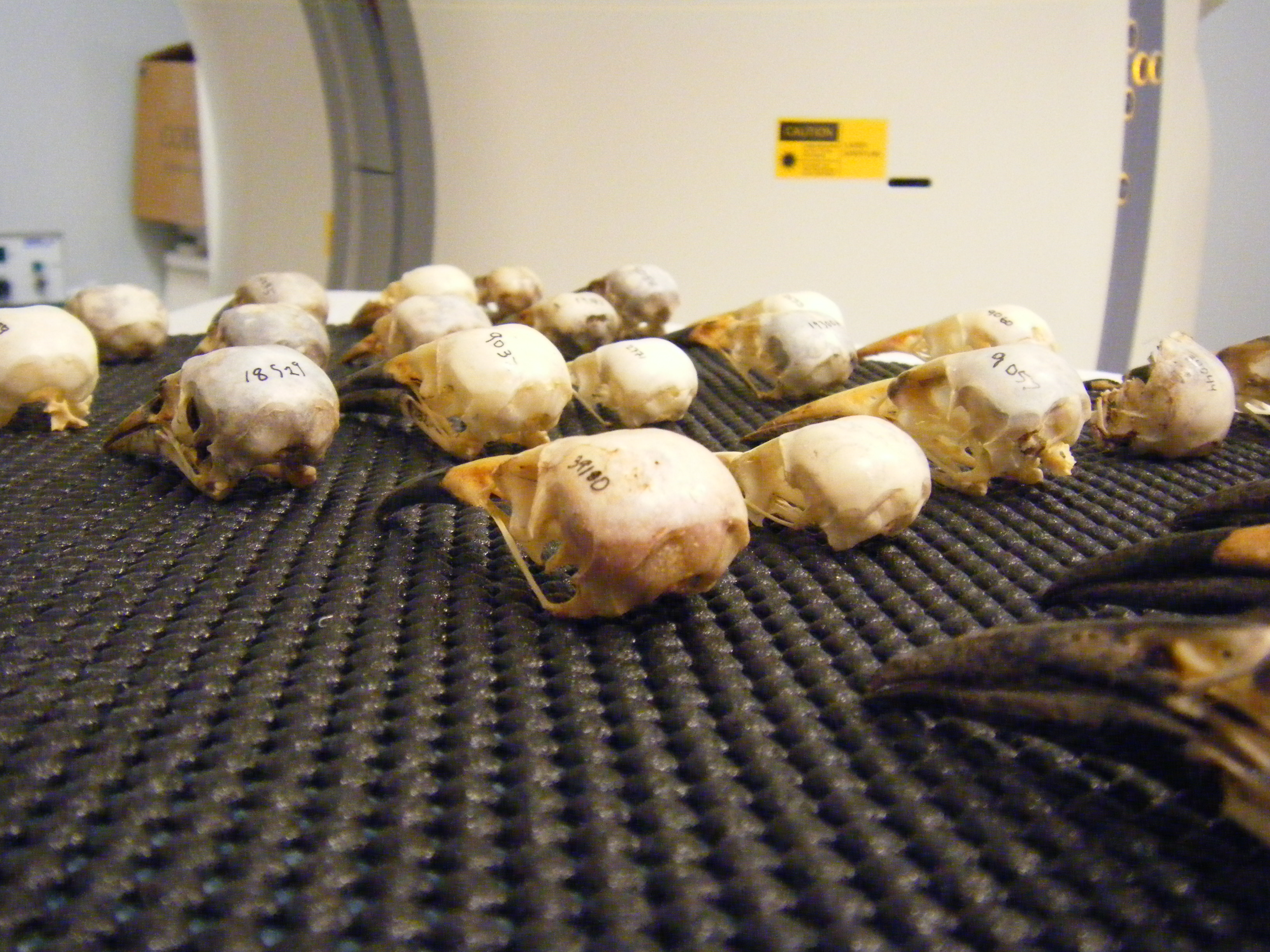

Supplement: Figure S1 — Grackle skulls on the CT scanner bed about to be scanned. [file peerj-03-1000-s001.jpg]

Image size: 512 x 512  
View size: 924 x 620  
WL: -97 WW: 2792

A

626675 ( 3 m , 19 d )  
Brain+Head Wo  
ABD\_WO  
7

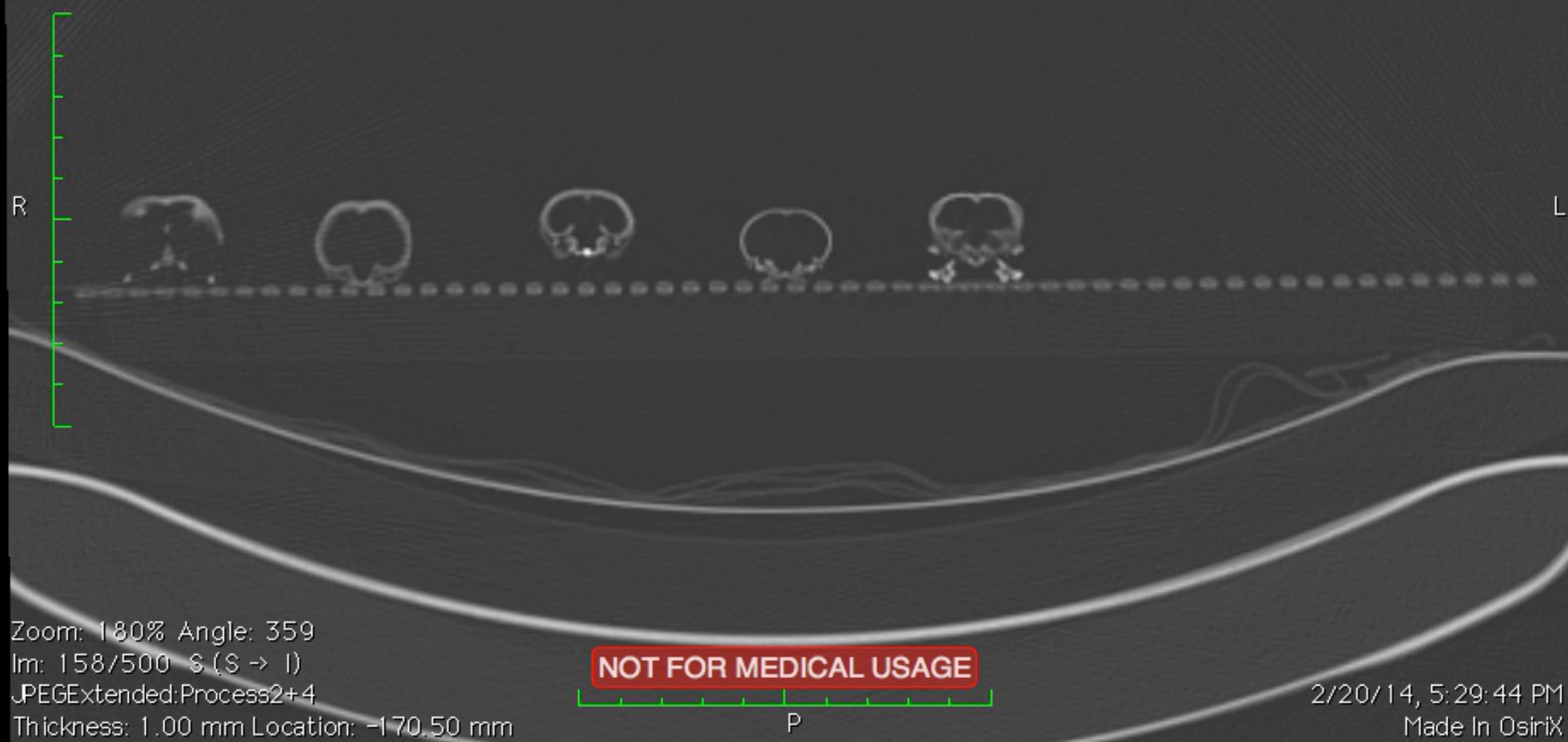

Supplement: Figure S2 — Screenshot of a CT scan showing five grackle skulls using the software OsiriX. [file peerj-03-1000-s002.pdf]
