# Supplementary material for: Can endocranial volume be estimated accurately from external skull measurements in great-tailed grackles (Quiscalus mexicanus)?
Source: PeerJ. 2015 Jun 11;3:e1000. doi: 10.7717/peerj.1000 (PMC4465945; doi:10.7717/peerj.1000)
Supplement: Table S1 — Archive data for each Quiscalus mexicanus skull measured (SBMNH, Santa Barbara Museum of Natural History; MSB, Museum of Southwestern Biology, KU, University of Kansas Biodiversity Institute and Natural History Museum). ∗, Quiscalus mexicanus. [file peerj-03-1000-s003.docx]

Supplemental Table S1. Archive data for each *Quiscalus mexicanus* skull measured (SBMNH=Santa Barbara Museum of Natural History, MSB=Museum of Southwestern Biology, KU=University of Kansas Biodiversity Institute and Natural History Museum). *=*Quiscalus mexicanus mexicanus*

| Institution | ID | Sex | Age | Location | Date Collected |
| --- | --- | --- | --- | --- | --- |
| SBMNH | 5069 | Female | Immature | Lake Los Carneros, CA | Aug 1997 |
| MSB | 11770 | *Male | Immature | Las Cruces, NM | Nov 1932 |
| MSB | 14127 | Male | Adult | Albuquerque, NM | Apr 1993 |
| MSB | 14285 | Male | Adult | Albuquerque, NM | May 1993 |
| MSB | 16749 | Female | Immature | Mesilla Park, NM | Jul 1969 |
| MSB | 17685 | Male | Adult | Phoenix, AZ | Jun 1969 |
| MSB | 17687 | Female | Immature | Nayarit, Mexico | May 1973 |
| MSB | 18529 | Male | Adult | Rio Rancho, NM | Jun 1994 |
| MSB | 19266 | Male | Immature | Artesia, NM | Sep 1992 |
| MSB | 19273 | Female | Adult | La Joya Game Refuge, NM | Jul 1992 |
| MSB | 19300 | Male | Adult | Logan, NM | Jun 1993 |
| MSB | 21956 | Female | Adult | Port Isabel, TX | Apr 1997 |
| MSB | 22394 | Male | Adult | Albuquerque, NM | Oct 1999 |
| MSB | 39044 | Male | Adult | Carlsbad, NM | Sep 2011 |
| MSB | 39180 | Male | Adult | Moriarty, NM | Nov 2001 |
| MSB | 6777 | Male | Adult | Albuquerque, NM | Apr 1990 |
| MSB | 7196 | Female | Immature | Artesia, NM | Jun 1990 |
| MSB | 8969 | Male | Adult | San Marcial, NM | Jul 1991 |
| MSB | 8970 | Male | Adult | San Marcial, NM | Jul 1991 |
| MSB | 9032 | Male | Adult | Albuquerque, NM | Jul 1991 |
| MSB | 9056 | Male | Immature | Garfield, NM | Apr 1991 |
| MSB | 9057 | Male | Adult | Garfield, NM | Apr 1991 |
| MSB | 9058 | Male | Immature | Garfield, NM | Apr 1991 |
| MSB | 9059 | Male | Immature | Garfield, NM | May 1991 |
| MSB | 9060 | Male | Immature | Garfield, NM | May 1991 |
| KU | 12300 | Female | Adult | Veracruz, Mexico | Mar 1949 |
| KU | 12301 | Female | Adult | Veracruz, Mexico | Mar 1949 |
| KU | 12302 | Female | Adult | Veracruz, Mexico | Mar 1949 |
| KU | 12303 | Female | Adult | Veracruz, Mexico | Mar 1949 |
| KU | 12304 | Female | Adult | Veracruz, Mexico | Mar 1949 |
| KU | 12305 | Female | Adult | Veracruz, Mexico | Mar 1949 |
| KU | 12306 | Female | Adult | Veracruz, Mexico | Mar 1949 |
| KU | 11602 | Female | Adult | Jalisco, Mexico | May 1950 |
| KU | 7767 | Female | Adult | Veracruz, Mexico | Oct 1947 |
| KU | 7576 | Female | Immature | Veracruz, Mexico | Sep 1947 |
| KU | 4239 | Female | Adult | Puebla, Mexico | Aug 1954 |
| KU | 550313-5 | Female | Adult | Guatemala | Mar 1955 |
| KU | 9022 | Female | Adult | Veracruz, Mexico | Mar 1948 |
| KU | 9023 | Female | Adult | Veracruz, Mexico | Mar 1948 |
| KU | 322 | Male | Adult | Hidalgo, TX | Apr 1967 |
